# Effect of Haloperidol and Olanzapine on Hippocampal Cells’ Proliferation in Animal Model of Schizophrenia

**DOI:** 10.3390/ijms23147711

**Published:** 2022-07-12

**Authors:** Jana Osacka, Alexander Kiss, Zuzana Bacova, Andrej Tillinger

**Affiliations:** Biomedical Research Center, Institute of Experimental Endocrinology, Slovak Academy of Sciences, Dúbravská Cesta 9, 845 05 Bratislava, Slovakia; ueenkiss@savba.sk (A.K.); zuzana.bacova@savba.sk (Z.B.); ueentill@savba.sk (A.T.)

**Keywords:** neurogenesis, haloperidol, olanzapine, subventricular zone, hippocampus

## Abstract

Aberrant neurogenesis in the subventricular zone (SVZ) and hippocampus (HIP) contributes to schizophrenia pathogenesis. Haloperidol (HAL) and olanzapine (OLA), commonly prescribed antipsychotics for schizophrenia treatment, affect neurogenesis too. The effect of HAL and OLA on an mHippoE-2 cell line was studied in vitro where we measured the cell number and projection length. In vivo, we studied the gene expression of *DCX*, *Sox2*, *BDNF*, and *NeuN* in the SVZ and HIP in an MK-801-induced animal schizophrenia model. Cells were incubated with HAL, OLA, and MK-801 for 24, 48, and 72 h. Animals were injected for 6 days with saline or MK801 (0.5 mg/kg), and from the 7th day with either vehicle HAL (1 mg/kg) or OLA (2 mg/kg), for the next 7 days. In vitro, HAL and OLA dose/time-dependently suppressed cells’ proliferation and shortened their projection length. HAL/OLA co-treatment with MK-801 for 24 h reversed HAL’s/OLA’s inhibitory effect. In vivo, HAL and OLA suppressed *DCX* and *NeuN* genes’ expression in the HIP and SVZ. MK-801 decreased *DCX* and *NeuN* genes’ expression in the HIP and OLA prevented this effect. The data suggest that subchronic HAL/OLA treatment can inhibit *DCX* and *NeuN* expression. In an MK-801 schizophrenia model, OLA reversed the MK-801 inhibitory effect on *DCX* and *NeuN* and HAL reversed the effect on DCX expression; however, only in the HIP.

## 1. Introduction

Schizophrenia is considered to be a neurodevelopmental and neurodegenerative disease. Knowledge of the adult neurogenesis in schizophrenia is poor; nevertheless, the aberrant neurogenesis is supposed to contribute to its pathogenesis, pathophysiology, and symptoms [1]. Available studies indicate abnormalities in the generation of mature neurons from neural stem cells, consistent with reduced adult neurogenesis in animal models [2] and the neurodevelopmental hypothesis of schizophrenia [3]. Neurogenesis occurs throughout the life of rodents in specific areas of the brain including the subventricular zone (SVZ) of the lateral ventricles and the subgranule zone of the hippocampus (HIP) [4]. Recently, adult neurogenesis has also been evidenced in other brain regions including the hypothalamus, where neurogenesis occurs mainly along and beneath the third ventricle wall. However, the level of neurogenesis appears to be lower in the hypothalamus in comparison with the two canonical neurogenic regions, i.e., the SVZ and HIP [5,6]. Previous studies have shown that neurogenesis can be affected by numerous stimuli [7,8] including antipsychotics.

Antipsychotics, drugs ordinarily used in the treatment of schizophrenia, are commonly divided into typical and atypical ones. Typical antipsychotics are effective only against positive symptoms of schizophrenia, while atypical antipsychotics are effective against negative and cognitive symptoms as well [9]. Antipsychotics can modulate dysfunction in the chemical neurotransmission that plays an important role in reducing the symptoms of schizophrenia, but also in their potential role in neuroprotection and neurotoxicity. The neuroprotective effect of antipsychotics can be involved in their therapeutic properties such as cognitive enhancement or the prevention of disease progression and clinical deterioration. The neurotoxic effect of antipsychotics may be related to side effects’ development [10].

Recent research has demonstrated that while typical antipsychotics are associated with multiple neurotoxic effects, atypical ones are neuroprotective [11]. The mechanisms, by which atypical antipsychotics may exert neuroprotection, include neurogenesis, protection against toxicity, ischemia, and insults or the upregulation of neurotrophic factors such as brain-derived neurotrophic factor (BDNF) and nerve growth factor (NGF). The increase in neurotrophic factors induced by atypical antipsychotics goes along with studies that have demonstrated an increase in neuroplasticity, neurogenesis, the repair of dendritic spine changes, proliferation of SVZ neurons, and preservation of brain volume in schizophrenia subjects [12]. The extent of atypical antipsychotics’ induced neuroprotection seems to be dose-dependent with more neuroprotection efficacy when applied in higher doses.

Haloperidol (HAL) represents a typical antipsychotic commonly used in the treatment of mental health states with a risk–benefit profile comprising extrapyramidal side effects. There is a lot of evidence indicating that HAL can dose-dependently exert a toxic effect in both in vitro and in vivo conditions [10,13,14]. On the other hand, olanzapine (OLA) is an atypical antipsychotic drug widely used in the treatment of schizophrenia patients due to its low incidence of extrapyramidal symptoms [15]. Previous in vitro and in vivo studies have demonstrated that OLA may exert a neuroprotective effect under specific conditions [16,17].

Postmortem studies examining the brains of schizophrenic patients have shown changes in the density and expression of immature neuron markers and trophic factors including BDNF [1]. Knowledge about the effect of antipsychotics on adult neurogenesis provides little clear data about its influence on adult neurogenesis during schizophrenia treatment. Therefore, the aim of the present study was to investigate the effect of haloperidol and olanzapine on the neurogenesis in (1) in vitro conditions, i.e., on the quantity of cells and their projection length in a mouse hippocampal mHippoE-2 (CLU196) cell line and (2) in vivo in the SVZ and HIP in the animal model of schizophrenia. In vivo, we studied gene expression and immunoreactivity for *DCX*, *Sox2*, *BDNF*, and *NeuN*, i.e., markers expressed during different stages of neurogenesis. *Sox2* is a stem cell marker, *DCX* is an immature neuronal marker, and *NeuN* is the most widely used indicator for “mature neurons” [18].

## 2. Results

### 2.1. In Vitro Studies

#### 2.1.1. Proliferation and Neuronal Viability

We counted the number of cells after 24 h, 48 h, and 72 h incubation with HAL (0.1 µM, 10 µM), OLA (0.1 µM, 10 µM), MK-801 (20 µM), MK-801 + HAL (20 µM + 10 µM), and MK-801 + OLA (20 µM + 10 µM). For every time interval we calculated the percentage of cells compared to the controls that represented 100%.

**Figure 1:**
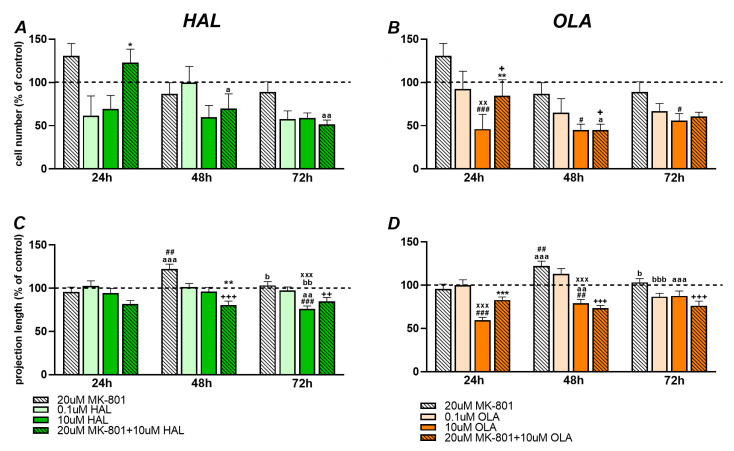
Cell number (**A**,**B**) and length of cells’ projection (**C**,**D**) after 20 µM MK-801, 0.1 and 10 µM HAL/OLA treatment, and 20 µM MK-801 + 10 µM HAL/OLA co-treatment after 24 h, 48 h, and 72 h. Data are expressed as percentage compared to controls that represent 100% (dotted line in graphs). ^#^ *p* < 0.05 vs. control, ^##^ *p* < 0.01 vs. control, ^###^ *p* < 0.001 vs. control, * *p* < 0.05 vs. 10 µM HAL/OLA, ** *p* < 0.01 vs. 10 µM HAL/OLA, *** *p* < 0.001 vs. 10 µM HAL/OLA, ^xx^ *p* < 0.01 vs. 0.1 µM HAL/OLA, ^xxx^ *p* < 0.001 vs. 0.1 µM HAL/OLA, ^+^ *p* < 0.05 vs. 20 µM MK-801, ^++^ *p* < 0.01 vs. 20 µM MK-801, ^+++^ *p* < 0.001 vs. 20 µM MK-801, ^a^ *p* < 0.05 vs. the same treatment 24 h, ^aa^ *p* < 0.01 vs. the same treatment 24 h, ^aaa^ *p* < 0.001 vs. the same treatment 24 h, ^b^ *p* < 0.05 vs. the same treatment 48 h, ^bb^ *p* < 0.01 vs. the same treatment 48 h, ^bbb^ *p* < 0.001 vs. the same treatment 48 h.

The treatment of the mHippoE-2 cell line with antipsychotics affected the number of cells as shown by two-way ANOVA analysis: for HAL (F(3, 71) = 3.16, *p* = 0.031), for OLA (F(3, 70) = 7.59, *p* < 0.001), and for MK-801 (F(2, 53) = 7.08, *p* = 0.002). Incubation with MK-801 + HAL for 24 h significantly increased the number of cells compared to 10 µM HAL (*p* = 0.016). Noticeably, 48 h and 72 h incubation with MK-801 + HAL decreased the number of cells when compared with the 24 h one (*p* = 0.017 and *p* = 0.002, Figure 1A).

The number of cells were decreased by 10 µM OLA compared to the controls for all three time intervals (*p* = 0.001 for 24 h, *p* = 0.015 for 48 h, and *p* = 0.043 for 72 h, Figure 1B) and by 0.1 µM OLA after 24 h incubation (*p* = 0.003, Figure 1B). The co-treatment of MK-801 + OLA reversed the inhibitory effect of 10 µM OLA on the cell number but only after 24 h incubation (*p* = 0.008, Figure 1B). The incubation of cells with MK-801 + OLA for 24 h and 48 h suppressed the number of cells compared to the MK-801 treatment (*p* = 0.035 and *p* = 0.026, Figure 1B). After 48 h, MK-801 + OLA co-treatment also lowered the amount of cells in comparison with that after 24 h (*p* = 0.044, Figure 1B).

#### 2.1.2. Projection Length

Projection length is also expressed as a percentage compared to the controls that represented 100%. Treatment of the mHippoE-2 cell line with MK-801 and antipsychotics for different time intervals (24 h, 48 h, and 72 h) affected the length of the cells’ projections as shown by two-way ANOVA analysis: HAL (F(3, 691) = 11.25, *p* < 0.001), OLA (F(3, 587) = 22.55, *p* < 0.001), and MK-801 (F(2, 532) = 20.67, *p* < 0.001), OLA and time interaction (F(6, 587) = 4.34, *p* < 0.001), and MK-801 and time interaction (F(4, 532) = 2.78, *p* = 0.026).

**Figure 2:**
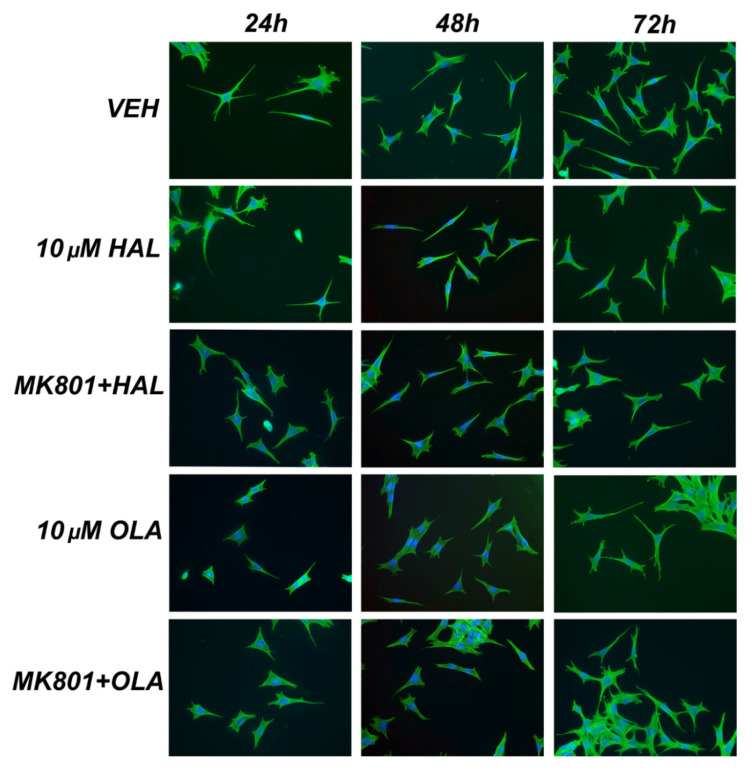
Representative fluorescent microscopic images of mHippoE-2cells after 24 h, 48 h, and 72 h incubation with vehicle, 10 µM HAL/OLA, and 20 µM MK-801 + 10 µM HAL/OLA. Double immunofluorescence for nuclei stained by DAPI (blue) and actin filaments stained by phalloidin-iFluor (green). Magnification × 400.

The projections of cells incubated with 10 µM HAL for 72 h were shorter compared to the controls, 0.1 µM HAL (*p* < 0.001 for both, Figure 1C), and those incubated for 24 h and 48 h (*p* = 0.01 and *p* = 0.004, respectively, Figure 1C and Figure 2). Co-treatment of 10 µM HAL with MK-801 (i.e., MK-801 + HAL) decreased the projections’ length after 48 h in comparison with 10 µM HAL (*p* = 0.008, Figure 1C and Figure 2).

Cells treated with 0.1 µM OLA for 24 and 48 h had longer projections than cells incubated with 10 µM OLA for the same time intervals (*p* < 0.001 for both, Figure 1D), and also for those treated for 48 h in comparison with those treated for 72 h (*p* = 0.001, Figure 1D). Treatment with 10 µM OLA for 24 h and 48 h shortened the cells’ projection compared to the controls (*p* < 0.001 and *p* = 0.002, respectively, Figure 1D and Figure 2), and 24 h treatment also shortened the projections in comparison with 48 h and 72 h treatments (*p* = 0.003 and *p* < 0.001, respectively, Figure 1D and Figure 2). Co-treatment with MK-801, i.e., MK-801 + OLA, suppressed the inhibitory effect of 10 µM OLA after 24 h (*p* < 0.001, Figure 1D and Figure 2).

Incubation with 20 µM MK-801 for 48 h prolonged the projections compared to the controls (*p* = 0.003, Figure 1C,D), and the 24 h and 72 h incubations (*p* < 0.001 and *p* = 0.016, respectively, Figure 1C,D). Cells co-treated with MK-801 + HAL or MK-801 + OLA for 48 h and 72 h exhibited shorter cell projections than those treated with MK-801 (*p* < 0.001 and *p* = 0.003 for HAL, respectively, and *p* < 0.001 for both time intervals for OLA, Figure 1C,D).

### 2.2. Genes’ Expression

#### 2.2.1. *DCX* mRNA

**Figure 3:**
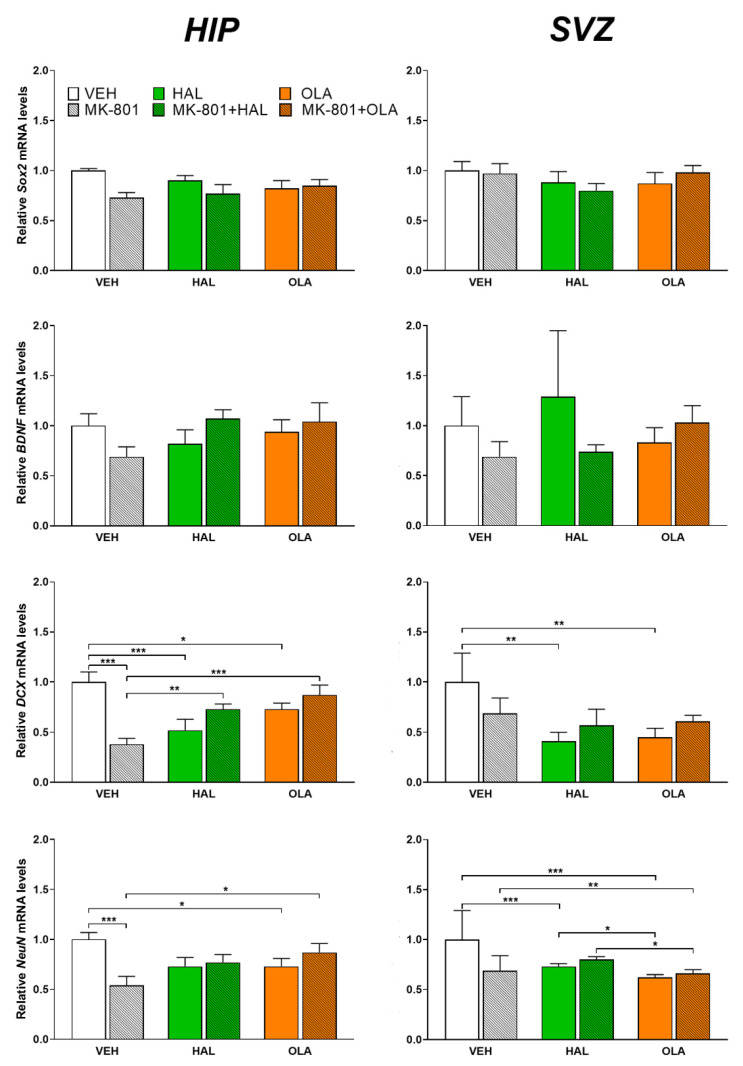
Relative *Sox2*, *BDNF*, *DCX*, and *NeuN* mRNA levels in the HIP and SVZ. Data are presented as fold change relative to control, taken as 1 (mean ± SEM, n = 7 animals pre-group). * *p* < 0.05, ** *p* < 0.01, *** *p* < 0.001.

In the HIP there was an effect of the MK-801 and antipsychotic treatment on the *DCX* mRNA level as shown by two-way ANOVA analysis (F(2, 41) = 17.12, *p* < 0.001). Both antipsychotics, HAL and OLA, suppressed *DCX* mRNA levels in the saline-treated animals (*p* < 0.001 and *p* = 0.034, respectively, Figure 3). The MK-801 group had a lower *DCX* mRNA level compared to the VEH one (*p* < 0.001, Figure 3). In addition, the MK-801 + HAL and MK-801 + OLA groups exhibited higher *DCX* gene expression than the MK-801 one (*p* = 0.002 and *p* < 0.001, respectively, Figure 3).

The impact of the MK-801 and antipsychotic treatment on the *DCX* mRNA level (F(2, 39) = 3.31, *p* = 0.049) was revealed by two-way ANOVA, as well as in the SVZ. HAL and OLA suppressed *DCX* mRNA in comparison with the VEH group (*p* = 0.003 and *p* = 0.005, respectively, Figure 3).

#### 2.2.2. *NeuN* mRNA

The effect of the MK-801 and antipsychotic treatment on the hippocampal *NeuN* mRNA level was shown by two-way ANOVA analysis (F(1, 41) = 6.42, *p* = 0.004). The VEH group had a higher *NeuN* mRNA level in comparison with the OLA and MK-801 (*p* = 0.035 and *p* < 0.001, respectively, Figure 3) groups. The MK801 + OLA group exhibited higher *NeuN* gene expression than the MK-801 group (*p* = 0.012, Figure 3).

In the SVZ, two-way ANOVA proved the suppressing influence of both antipsychotics on the *NeuN* mRNA levels (F(2, 40) = 18.83, *p* < 0.001). The HAL and OLA experimental groups exhibited decreased *NeuN* gene expression in comparison with the VEH group (*p* < 0.001 for both, Figure 3), and also for OLA in comparison with the HAL one (*p* = 0.048, Figure 3). The MK-801 + OLA group had lower *NeuN* mRNA levels than the MK-801 (*p* = 0.006, Figure 3) and MK-801 + HAL groups (*p* = 0.032, Figure 3).

#### 2.2.3. *Sox2* mRNA

The MK-801 and HAL/OLA treatment did not affect the *Sox2* mRNA levels either in the HIP or SVZ (Figure 3).

#### 2.2.4. *BDNF* mRNA

The MK-801 and HAL/OLA treatment did not affect the *BDNF* mRNA levels either in the HIP or SVZ (Figure 3).

### 2.3. Immunofluorescence

**Figure 4:**
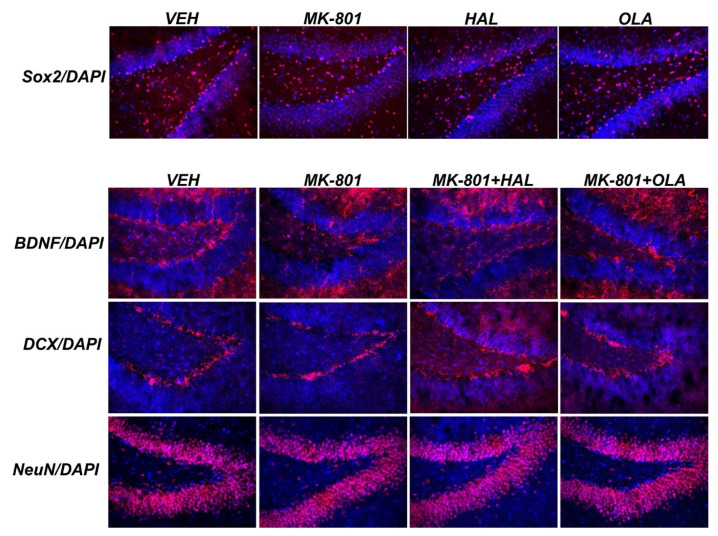
Representative immunofluorescent pictures show staining with anti-Sox2, anti-BDNF, anti-DCX, and anti-NeuN antibodies (red) and with DAPI for nuclei (blue) in the hippocampal part, gyrus dentatus. Magnification × 200.

Expression of Sox2, BDNF, DCX, and NeuN in the hippocampal part, the gyrus dentatus (GD), and SVZ was examined using immunofluorescence. Sox2 was expressed mainly in the subgranular zone and the polymorph layer of the GD. MK-801, HAL, and OLA treatment slightly decreased the amount of Sox2 positive cells, especially in the subgranular zone of the GD (Figure 4). BDNF immunopositive cells were located mainly in the subgranular zone of the GD. MK-801 seemed to decrease the BDNF immunoreactivity, and HAL/OLA seemed to suppressed this effect (Figure 4). DCX immunopositive cells were restricted to the subgranular zone of the GD. MK-801, HAL, and also OLA treatment decreased the amount of DCX immunoreactive cells in comparison with the VEH experimental group. In the MK-801 + HAL and MK-801 + OLA groups, we detected slightly more DCX immunopositive cells in comparison with the MK-801 group (Figure 4). NeuN immunoreactive cells were located mainly in the granular layer of the GD. MK-801 seemed to decrease the number of NeuN immunopositive cells in the upper part of the GD when compared with the controls, but we did not notice marked differences among the other experimental groups (Figure 4). In the SVZ, the density of immunostained markers was overloaded, and no differences among the individual groups were recognizable (data not shown).

## 3. Discussion

The present study showed that in in vitro conditions, HAL and OLA dose- and time-dependently suppressed the cells’ proliferation (10 µM OLA exhibited the most pronounced effect) and shortened the length of the cells’ projections (10 µM HAL and 10 µM OLA) in the mHippoE-2 cell line. Co-treatment of HAL/OLA with MK-801 reversed HAL’s/OLA’s inhibitory effect but only after 24 h incubation (MK-801 + HAL did not affect the length of the cells’ projection). In in vivo conditions, the inhibitory effect of HAL and OLA on the expression of *DCX* gene (a marker of neuronal progenitor cells and immature neurons) was observed in the HIP and SVZ. OLA treatment also decreased the *NeuN* gene (a marker of postmitotic neurons) level in both regions of interest, whereas with HAL treatment this occurred only in the SVZ. MK-801 treatment suppressed the expression of *DCX* and *NeuN* genes in the HIP. However, OLA was able to prevent this effect, while HAL only in the case of *DCX*. No protective effect of HAL/OLA was observed in the SVZ.

Data from in vitro studies have emphasized the dose- and time-dependent effect of antipsychotics on the cells. Most of the studies have demonstrated the cytotoxic effect of HAL leading to cell apoptosis [13] and the reduction in MTT metabolism [14]. A dose-dependent cytotoxic effect of OLA and HAL (HAL being more cytotoxic) has also been shown in human dopaminergic neuroblastoma cell line SH-SY5Y [17]. On the other hand, it was shown that certain doses of OLA had a neuroprotective effect [17,19]. Moreover, 96 h after the treatment, they noticed cells’ recovery [17]. In the present study, 10 µM OLA (10 µM HAL only after 72 h) suppressed the cells’ proliferation, but the shortening of the cells’ projections was achieved by both antipsychotics. After 72 h incubation, 10 µM HAL, but not 10 µM OLA, shortened the cells’ projections that could indicate their recovery.

In in vitro conditions MK-801 dose-dependently induced a cytotoxic effect at the SH-SY5Y cells. The application of 25 and 50 μM MK-801 resulted in slight damage to the cells with neurites retracting and disappearing, while 100 and 200 μM MK-801 resulted in obvious cell damage including a collapsed network and increased cell debris [20]. However, in the present study we used 20 μM MK-801, which might be too low a concentration to induce a significant cytotoxic effect. Notably, after 24 h of the cells incubation with MK-801 together with HAL/OLA, MK-801 suppressed the inhibitory effect of the antipsychotic on the cells’ viability and the projection length, which disappeared after 48 and 72 h. These data suggest that the 24 h combination of 20 μM MK-801 with 10 µM HAL/OLA aroused the protective effect against HAL/OLA. A previous study has shown the interaction between the 5-HT and NMDA receptors and the different effects of the 5-HT7 receptors on NMDA receptor activity after acute and long-lasting activations [21]. Based on these data, we suggest that 5-HT receptors may play a role in the effect of HAL/OLA and MK-801 on cell viability and their projection lengths.

Accumulating evidence supports the importance of glutamatergic NMDA receptors’ hypofunction in schizophrenia. Since aberrant glutamate neurotransmission in animal models of schizophrenia can be attained by the administration of NMDA receptor antagonists including MK-801 [22], the MK-801 animal schizophrenia model was chosen for our study. In addition, positive, negative, and cognitive schizophrenia symptoms are manifested in this model. Impaired neurogenesis, i.e., the decreased proliferation of adult neural stem cells in the GD, has been demonstrated in animal schizophrenic models as well as in schizophrenic patients [23]. Neurogenesis has been studied via changes in the levels of neuronal differentiation markers such as *DCX* and *NeuN* in neurogenic regions of the adult rat brain including the subgranular zone of the GD and the SVZ of the lateral ventricles [24]. In our study of an MK-801-induced schizophrenia animal model, we observed decreased *DCX* and *NeuN* gene expression in the HIP, which was also confirmed by the DCX immunohistochemical data. *Sox2* and *BDNF* levels were also lower but not statistically significant. Our finding is in accordance with previously published data that have also shown, after MK-801 treatment, a significant decrease in the DCX and NeuN immunoreactive cells’ number in the HIP [25,26] but no change in the *BDNF* mRNA level [27]. GD-NR1 KO mice (NR1 is a glycine-binding NMDA receptor subunit) also showed impairment in neurogenesis during postnatal development and adulthood and a significant reduction in the density of DCX positive cells in the GD [28]. However, we did not observe a significant inhibitory effect of MK-801 on any of the markers studied in the SVZ.

Previous studies have shown that typical and atypical antipsychotics may affect the neurogenesis differently. While HAL had no effect on the proliferation or cell survival in the GD, OLA markedly increased the number of bromodeoxyuridine (BrdU) positive cells in the HIP [4,29]. The effect of the atypical antipsychotics appears to be dependent on the type, dosage, and time period of the treatment [4]. Chronic (21-day) OLA administration in a dose of 2 mg/kg increased the number of BrdU-labeled cells in the GD, while its subchronic (7 days) administration had no effect on cell proliferation. However, neither the 7- nor the 21-day treatments affected the number of BrdU-labeled cells in the SVZ [30]. Another study showed that a 4-week treatment with OLA (10 mg/kg) induced a proneurogenic effect and increased the number of Sox2 and DCX positive cells in the SVZ but not in the GD [31]. In the hypothalamus, a single injection of OLA (5 mg/kg)/HAL (2 mg/kg) suppressed the DCX immunohistochemistry, while chronic OLA treatment had no effect [32]. In our study, a 7-day treatment suppressed *DCX* and *NeuN* mRNA levels after HAL and OLA treatments in the SVZ and HIP (HAL did not affect the *NeuN* level in the HIP). The available data indicate that subchronic/short-term treatments with both typical and atypical antipsychotics seem to inhibit neurogenesis under physiological conditions. This effect of HAL might be mediated via dopaminergic D2 receptors. Takamura et al. (2014), after the 21-day treatment of animals with D2 receptors antagonist sulpiride, did not observe any effect on the proliferation of the adult rat GD-derived neural precursor cells [33]. However, according to the above mentioned data, we can speculate about the different effects of D2 receptors antagonism on cell proliferation/survival depending on time. OLA has a higher affinity for serotonergic 5-HT2A than dopaminergic D2 receptors. Serotonin is known to influence cell proliferation and the survival of newly generated neurons. Acute and 7-day treatment with the 5-HT2 receptor agonist (α-methyl-5-HT) has been shown to decrease the number of BrdU positive cells, which were BrdU/nestin and also BrdU/nestin/DCX positive, which goes along with our findings [34]. We did not observe any effect of either HAL or OLA on the *BDNF* or *Sox2* mRNA levels in the SVZ or HIP. In the case of HAL, previously published data also did not show any effect on *BDNF* mRNA levels in the HIP [35,36], but long-term OLA treatment increased *BDNF* gene expression in the HIP and the number of Sox2 positive cells in the SVZ [31,35]. We assume that the 7-day OLA treatment was not sufficient time to induce such changes.

As we mentioned above, MK-801 treatment decreased the expression of *DCX* and *NeuN* genes in the HIP. Both HAL and OLA reversed the inhibitory effect of MK-801 (in the case of *NeuN*, only OLA). The protective effect of some antipsychotics on symptoms and anomalies observed in animal models of schizophrenia have already been published. OLA was able to reverse MK-801-induced psychosis-like behavior in mice [37] and OLA, but not HAL, significantly increased the number of BrdU-labeled cells in MK-801-treated mice [38]. Maeda et al. (2007) observed a decreased neural stem cell proliferation in the GD of a phencyclidine mice model of schizophrenia; however, HAL was not effective in preventing the impairment of the neural stem cells’ proliferation [39]. We showed that HAL was able to suppress the inhibitory effect of MK-801 on *DCX*. Previous studies have shown that dopamine positively regulates cell proliferation, and proliferation decreases after dopamine depletion can be reversed by dopamine stimulation in vivo [40]. Dopamine is able to modulate NMDA receptor activity, and D2 receptors have been shown to reduce NMDA receptor activity and neuronal calcium influx [41]. This could be a possible mechanism that might reverse the MK-801 suppressive effect on DCX gene expression.

OLA has a more complex pharmacological profile than HAL and assigns binding affinities for a number of neurotransmitter receptors, including the serotonergic ones. The literature data indicate that 5-HT2A receptors may modulate NMDA receptor signaling via a different mechanism mediated by multiple proteins of the postsynaptic density, the core components of the NMDA receptors’ network [42]. We observed this protective effect of OLA only in the HIP. It is noteworthy that in the SVZ we observed the opposite effect of OLA on *NeuN* expression. OLA even potentiated the MK-801 inhibitory effect on the *NeuN* gene’s expression. Our data indicate that the effect of MK-801 and OLA on the neurogenesis in the SVZ and HIP might be mediated by different pathways. Notably, HAL did not reverse the inhibitory effect of MK-801 on *NeuN* expression in the HIP. It seems that HAL is able to avert the MK-801 effect on newly formed (DCX positive), but not surviving cells (NeuN positive), only in the HIP.

In summary, our data demonstrate that under physiological conditions, HAL in a dose of 1 mg/kg and OLA in a dose of 2 mg/kg, inhibited the expression of *DCX* and *NeuN* genes. However, in an MK-801-induced schizophrenia animal model, OLA reversed the inhibitory effect of MK-801 on *DCX* and *NeuN* and HAL on *DCX* only in the HIP.

## 4. Materials and Methods

### 4.1. In Vitro Experiments

#### 4.1.1. Cell Culture

Embryonic mouse hippocampal cell line mHippoE-2 (CLU196) immortalized by retroviral transfer of SV40 T-Ag was obtained from Cedarlane (Burlington, ON, Canada). Cells were cultured in Dulbecco’s Minimum Essential Medium with 4500 mg/L glucose, supplemented with 2 mM L-glutamine, 100 U/mL penicillin, 100 μg/mL streptomycin (Biosera, Nuaille, France), and 10% HyClone Fetal Bovine Serum (Cytiva, Marlborough, MA, USA) and maintained in humidified atmosphere containing 5% CO_2_ at 37 °C. Passaging was performed after the cells became confluent by gentle trypsinization.

#### 4.1.2. Cell Counting

Hippocampal mHippoE-2cells were cultured at a density of 10^4^ cells/mL in a 24-well plate (TPP, Trasadingen, Switzerland) containing glass cover slips (Heathrow Scientific, Vernon Hills, IL, USA). Cells were grown in the presence or absence of HAL (0.1 and 10 µM), OLA (0.1 and 10 µM), MK-801 (20 µM), or combination of 20 µM MK-801 +10 µM HAL/OLA for 24, 48, and 72 h. Neuronal proliferation was evaluated by cell counting with the help of hemocytometer Bürker Chamber.

#### 4.1.3. Projection Length

Hippocampal mHippoE-2cells were cultured at a density of 10^4^ cells/mL in a 24-well plate (TPP, Trasadingen, Switzerland) containing glass cover slips (Heathrow Scientific, Vernon Hills, IL, USA). Cells were grown in the presence or absence of HAL (0.1 and 10 µM), OLA (0.1 and 10 µM), MK-801 (20 µM), or combination of 20 µM MK-801 + 10 µM HAL/OLA for 24, 48 and 72 h. The cells were washed with 1 mL cold phosphate-buffered saline (PBS) and subsequently fixed using 200 μL of 4% paraformaldehyde (Sigma-Aldrich, Steinheim, Germany) for 15 min at room temperature (RT). The fixed cells were washed three times with PBS and permeabilized by 200 μL PBS containing 0.1% Triton X-100 (Sigma-Aldrich, Steinheim, Germany) for 5 min. The cells were washed and stained. The actin filaments were stained by addition of 20 μL of Phalloidin-iFluor 488 Reagent (ab176753, Abcam, Cambridge, UK) directly on the cells without access to light for 40 min. Nuclei were stained by addition of 1 mL of 300 nM 4,6-diamidino-2-phenylindole (DAPI; Thermo Fisher Scientific, Waltham, MA, USA) for 1 min. Cells were observed using Zeiss Axio Imager A1 (Carl Zeiss, Oberkochen, Germany). Photographic images were taken from at least four random fields per cover slip. At least three cover slips were evaluated per experimental group. Projection outgrowth was determined by manually tracing the length of the longest neurite per cell (using ImageJ software) for all cells in a field that had an identifiable neurite and for which the entire neurite arbor could be visualized. Length of the neurite was measured from the edge of nucleus to the apical end of the projection.

### 4.2. In Vivo Experiments

Male Sprague Dawley rats (n = 66, 7–9 weeks old, weighing 270–290 g) were purchased from Charles River (Germany). Animals were housed 3–4 per cage in an animal facility with controlled temperature (22 ± 1 °C), 12-h light/dark cycle with lights on at 06:00 h and humidity 55%. Animals were provided with a regular rat chow (dry pellets) and tap water ad libitum. Principles of the Laboratory Animal Care and the experimental procedures used were approved by the State Veterinary and Food Administration of the Slovak Republic Committee (Approval protocol number 3203/18–221/3) and in accordance with the Council Directive 2010/63EU of the European Parliament and the Council of 22 September 2010 on the protection of animals used for scientific purposes.

#### 4.2.1. Experimental Design

**Figure 5:**
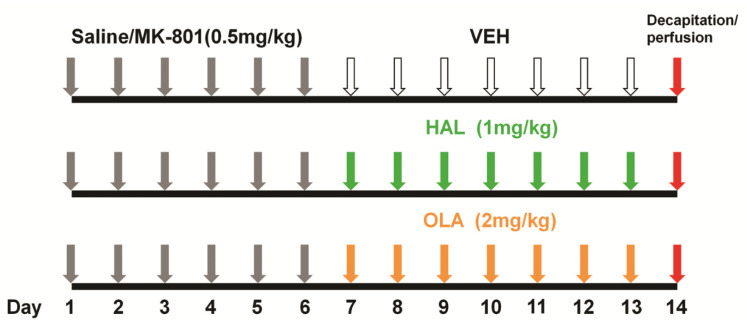
Time schedule of the experimental design.

The rats were acclimatized 7 days prior the experiment initiation. The animals were divided into 6 experimental groups: (1) saline + vehicle (VEH, n = 11); (2) MK-801 + vehicle (MK-801, n = 11); (3) saline + haloperidol (HAL, n = 11); (4) MK-801 + HAL (n = 11); (5) saline + olanzapine (OLA, n = 11); (6) MK-801 + OLA (n = 11). During the first six days of the experiment, animals were daily i.p. injected with saline (0.3 mL) or MK801 dissolved in saline (0.5 mg/kg). MK-801 is a noncompetitive NMDA receptor antagonist and is used to create animal model of schizophrenia with dysfunction of the glutamatergic system [43]. From the 7th day, for the following 7 days the animals were daily i.p. injected with vehicle (10% DMSO), HAL (1 mg/kg dissolved in vehicle), or OLA (2 mg/kg dissolved in vehicle) (Figure 5). During the whole experiment, the animals were regularly handled and weighed.

#### 4.2.2. Euthanasia of Animals, Tissue Processing

The rats were euthanized by decapitation (for gene expression analysis) or transcardial perfusion (for immunohistochemical analysis) 24 h after the last injection.

Following decapitation, the brains were carefully removed, frozen on dry ice, and kept at −72 °C for later biochemical analyses.

Four animals from each experimental group were sacrificed by transcardial perfusion. The rats were anesthetized and, afterwards, transcardially perfused with 60 mL of saline containing 450 μL of heparin (5000 IU/L, Zentiva, Bratislava, Slovakia) followed by 250 mL of fixative containing 4% paraformaldehyde in 0.1 M phosphate buffer (PB, pH 7.4). Removed brains were postfixed in a fresh fixative overnight, washed twice in 0.1 M PB, infiltrated with 30% sucrose for 2 days at 4 °C, cut into 30 μm thick coronal sections using cryostat (Reichert and Jung, Heidelberg, Germany), and collected in a cryoprotectant solution at −20 °C until further immunohistochemical processing.

#### 4.2.3. Microdissection of Brain Area

Brains from decapitated animals were acclimatized to −12 °C in a cryostat (Reichert and Jung, Germany) and sliced into 500-µm-thick coronal sections. These sections were placed on microscope slides. The brain areas, including SVZ (Bregma = 1.2 mm) and HIP (Bregma = −3.8 mm) were selected based on Paxinos and Watson (2007) brain atlas [44] and microdissected from the frozen sections by a punch technique [45] under stereomicroscope, using a special dissection needle. The dissected pieces of the brain tissue were collected in Eppendorf tubes, frozen in a liquid nitrogen, and stored at −75 °C until further analyses.

#### 4.2.4. RNA Isolation and Real-Time PCR

Total RNA was isolated using the TRI Reagent^®^RT (MRC, Inc., Cincinnatti, OH, USA) according to the manufacturers’ protocol and concentration was quantified using the NanoDrop 2000 (Thermo Fisher Scientific, Waltham, MA, USA). Reverse transcription of RNA (300 ng from each brain nucleus) was performed with the RevertAid H minusFirst Strand cDNA Synthesis kit (Thermo Fisher Scientific, Waltham, MA, USA) according to the manufacturer’s protocol, using an oligo dT primer. Semi-quantitative Real-Time PCR was set up in total volume of 25 μL containing 30 ng of template cDNA mixed with 12.5 μL of FastStart Universal SYBR Green Master Rox (Roche Diagnostics, Mannheim, Germany), 1 μL of specific primer pair set, and water. Sequences of specific primers are as follows: ***Sox2*** Forward 5′-ACAGCATGTCCTACTCGCAG-3′, Reverse 5′-A GTGGGAGGAAGAGGTAACCA-3′; ***BDNF*** Forward 5′-GCGCCCATGAAAGAAGCA AA-3′, Reverse 5′-TCGTCAGACCTCTCGAACCT-3′; ***DCX*** Forward 5′-ACGACCAAGAC GCAAATGGA-3′, Reverse 5′-ACAGTGGCAGGTACAAGTCC-3′; ***NeuN*** Forward 5′-CT TACGGAGCGGCACTGG-3′, Reverse 5′-CAAGAGAGTGGTGGGAACGC-3′; ***GAPDH*** Forward 5′-TGGACCACCCAGCCCAGCAAG-3′, Reverse 5′-GGCCCCTCCTGTTGTTA TGGGGT-3′. Each sample was analyzed on QuantStudio 5 Real-Time PCR System (Applied Biosystems, Waltham, MA, USA) under the following conditions: 1 cycle of 2 min at 50 °C followed by 1 cycle of 10 min at 95 °C and then 40 cycles of 95 °C for 15 s and 60 °C for 1 min. Data are normalized to GAPDH levels and expressed as the relative fold change, calculated using the ΔΔCt method [46]. Melting curve analysis was performed to confirm the specificity of the amplified products.

#### 4.2.5. Immunohistochemistry

Free-floating sections were washed 3 × 5 min in 0.1 M PB (pH = 7.4). For BDNF staining, sections were exposed to heat-mediated antigen retrieval in sodium citrate buffer (10 mM citrate pH 6 with 0.05% Tween-20) for 20 min at 95 °C. Afterwards they were left to cool down at RT for 20 min, washed 3 × 5 min in PB, incubated in the blocking solution (0.1 M PB with 3% NGS and 2% BSA) for 1 h at RT, and consequently with primary antibody. All primary antibodies were diluted in PB containing 4% NGS, 1% Triton X-100 and 0.1% sodium azide. Primary antibodies were diluted as follows: anti-Sox2 (rabbit, 1:500, AB5603, Millipore, Burlington, MA, USA), anti-BDNF (rabbit, 1:200, ab108319, Abcam, Cambridge, UK), anti-DCX (rabbit, 1:500, ab18723, Abcam, Cambidge, UK), anti-NeuN (rabbit, 1:1000, ab177487, Abcam, Cambridge, UK). Sections were incubated with primary antibodies for 24 h (Sox-2) or 48 h at 4 °C. Next, the sections were washed 3 × 5 min in PB and incubated for 2 h at RT with secondary antibodies in the dark. For immunofluorescence Alexa Fluor 555 secondary antibodies were all used at a concentration of 1:300 (Thermo Fisher Scientific, Waltham, MA, USA). Afterwards sections were again washed 3 × 5 min in 0.1 M PB, and after washings, nuclei were stained by addition of DAPI (1:1000, Thermo Fisher Scientific, Waltham, MA, USA) for 30 min at RT. Finally, the sections were washed 3 × 5 min in PB, mounted on adhesive slides, and coverslipped with Fluoromont (Thermo Fisher Scientific, Waltham, MA, USA) as anti-fading agent. The sections were visualized using Zeiss Axio Imager A1 and AxioCam ERc 5s camera (Carl Zeiss, Oberkochen, Germany).

### 4.3. Statistical Analysis

All the data were analyzed with SigmaPlot 11.0 software (Systat Software, Inc., Chicago, IL, USA). Normal distribution of obtained data was checked by Shapiro–Wilks test. If groups were not with homogeneous variance, square root transform was applied. If the distribution of the data was still non-normal, the nonparametric Kruskal–Wallis analysis, followed by *post hoc* comparisons, was applied. In all other cases, factorial analyses of variance (two-way ANOVA) for factors pre-treatment (saline, MK-801) and treatment (VEH, HAL, OLA) followed by Fisher’s LSD post hoc test was used. Data are reported as mean ± SEM. Differences were considered significant at *p* < 0.05. The outliers were excluded if the data points ranged more than 1.5 interquartile below the first quartile or above the third quartile.

## Data Availability

The data presented in the current study are available upon request from the corresponding author.
